# Factors associated with pain in children with hypermobility – a pilot study

**DOI:** 10.1186/1546-0096-12-S1-P100

**Published:** 2014-09-17

**Authors:** Sue Maillard, C Liossi, D Schoth, R Howard, Suellen Walker

**Affiliations:** 1 Great Ormond Street Hospital, London, UK

## Objectives

To explore the relationships between the degree of musculoskeletal pain, pain associated with disability and quality of life are affected by having hypermobile joints.

## Methods

Young people aged between 8-14 were recruited from the rheumatology based Non-Inflammatory Musculoskeletal Pain Clinic at Great Ormond Street Hospital over a 12 month period. They were assessed using biomechanical measures (muscle strength and degree of hypermobility), sensory processing using Quantitative Sensory Testing and psychological measures of anxiety, depression and pain coping styles using validated questions. Full Ethics approval was granted.

## Results

30 children were recruited (18 female: 12 male); mean age 11.08 with 77% being Caucasian. The mean Beighton score was 6.79/9. All patients reported pain mainly affecting lower limbs with an average score of 49/100 VAS. Degree of hypermobility did not have any impact. Reduced muscle strength was associated with increased pain and reduced quality of life. Other measures were compared to the norms for healthy children. Children with hypermobility appeared to demonstrate increased depression, negative mood, anhedonia and increased anxiety. They demonstrated reduced quality of life specifically with school, emotional well being, physical health and psychosocially. The subjects also had reduced sensitivity to touch including hot and cold.

## Conclusion

The pain experienced by children with hypermobile joints is complex and includes biomechanical, sensory, psychological and social factors. This pilot study is planned to be expanded into a multi-centred project depending upon funding.

## Disclosure of interest

None declared.

